# 101 Labeled Brain Images and a Consistent Human Cortical Labeling Protocol

**DOI:** 10.3389/fnins.2012.00171

**Published:** 2012-12-05

**Authors:** Arno Klein, Jason Tourville

**Affiliations:** ^1^Department of Psychiatry and Behavioral Science, Stony Brook University School of MedicineStony Brook, NY, USA; ^2^Department of Psychiatry, Columbia UniversityNew York, NY, USA; ^3^Department of Speech, Language, and Hearing Sciences, Boston UniversityBoston, MA, USA; ^4^Center for Computational Neuroscience and Neural Technology, Boston UniversityBoston, MA, USA

**Keywords:** human brain, cerebral cortex, MRI, anatomy, parcellation, labeling, segmentation

## Abstract

We introduce the Mindboggle-101 dataset, the largest and most complete set of free, publicly accessible, manually labeled human brain images. To manually label the macroscopic anatomy in magnetic resonance images of 101 healthy participants, we created a new cortical labeling protocol that relies on robust anatomical landmarks and minimal manual edits after initialization with automated labels. The “Desikan–Killiany–Tourville” (DKT) protocol is intended to improve the ease, consistency, and accuracy of labeling human cortical areas. Given how difficult it is to label brains, the Mindboggle-101 dataset is intended to serve as brain atlases for use in labeling other brains, as a normative dataset to establish morphometric variation in a healthy population for comparison against clinical populations, and contribute to the development, training, testing, and evaluation of automated registration and labeling algorithms. To this end, we also introduce benchmarks for the evaluation of such algorithms by comparing our manual labels with labels automatically generated by probabilistic and multi-atlas registration-based approaches. All data and related software and updated information are available on the http://mindboggle.info/data website.

## Introduction

Labeling the macroscopic anatomy of the human brain is instrumental in educating biologists and clinicians, visualizing biomedical data, localizing brain data for identification and comparison, and perhaps most importantly, subdividing brain data for analysis. Labeled anatomical subdivisions of the brain enable one to quantify and report brain imaging data within brain regions, which is routinely done for functional, diffusion, and structural magnetic resonance images (f/d/MRI) and positron emission tomography data.

Labeled regions are important in and of themselves for use in characterizing the morphometry of the brain. Brain morphology measures have been used as biological markers to characterize schizophrenia (Cachia et al., 2008), early- vs. intermediate-onset bipolar disorder, as well as bipolar and unipolar depression (Penttilä et al., 2009; Mangin et al., 2010; Kempton et al., 2011), and may someday aid clinicians in the diagnosis and prediction of treatment response for neuropsychiatric disorders. A biomarker of disease is defined by its ability to distinguish between clinical and control populations. Distinguishing among groups requires that the variation of a suitable measure *within* each group is separable from the variation *between* groups. This can only be accomplished by establishing “normative” data – data that allow accurate characterization of the usual variation within each group. Thus a significant hurdle to discovering better biomarkers for patient-specific psychiatric medicine is the lack of normative data to compare against. For this, we would need to carefully label the anatomy of many normal, healthy brains.

Another important application of labeled brain images is to train, test, and evaluate automated registration, segmentation, parcellation, and labeling algorithms. We conducted the world’s most extensive brain image registration evaluation studies (Klein et al., 2009, 2010b), but this was made possible only because of the public availability of manually labeled brain image data. These studies guided our research and exposed the limitations of existing labeled data sets and labeling protocols. We need a greater number of consistently, comprehensively, and accurately labeled brain images to drive brain imaging methods development.

The human cerebral cortex is difficult to label due to the great anatomical variation in the cortical folds and difficulty in establishing consistent and accurate reference landmarks across the brain (Ono et al., 1990; Petrides, 2011). Accurate definitions for landmarks and label boundaries is important because they underlie our assumptions of correspondence across brain image data. Although there is no ground truth to measure the accuracy of anatomical assignments, it is common to measure consistency across human labelers (e.g., Caviness et al., 1996) and variability across co-registered landmarks (e.g., Lohmann and von Cramon, 2000). There is a tradeoff between accuracy and efficiency, however. It takes a human operator 2–3 days to manually label a single brain image of 1 mm^3^ resolution without any initial set of label candidates. Automated anatomical labeling can help to initialize the labeling and make the process more efficient, by registering the labels from a probabilistic atlas (Bilder et al., 2008) or multiple individually labeled atlases (Klein et al., 2005; Heckemann et al., 2006; Aljabar et al., 2009) to a brain image, or by using dedicated anatomical labeling software (Fischl et al., 1999; Cointepas et al., 2001; Klein and Hirsch, 2005). Care must then be taken to reduce the bias of the human editor to the initialized set of automated labels. Hence an accurate, reproducible labeling protocol is crucial.

There are volume-based and surface-based cortical labeling protocols for delineating regions on either cross-referenced slices through an image volume or on inflated or flattened surface meshes. Examples of volume-based cortical labeling protocols include those developed at the Center for Morphometric Analysis at the Massachusetts General Hospital (Caviness et al., 1996), the Montreal Neurological Institute (Petrides, 2011), UCLA’s Laboratory of Neuro Imaging^1^ (Bilder et al., 2008), as well as the IOWA (Crespo-Facorro et al., 2000), AAL (Delcroix et al., 2002), and BrainCOLOR^2^ (Klein et al., 2010a) protocols. The most popular surface-based human cortical labeling protocols are the Desikan–Killiany (DK; Desikan et al., 2006) and Destrieux protocols (Destrieux et al., 2010) used by the FreeSurfer brain analysis software (Dale et al., 1999; Fischl et al., 1999, 2002, 2001). It is very difficult to reconcile the differences between (Bohland et al., 2009) or compare the accuracy of volume-based and surface-based labeling protocols or algorithms due to interpolation artifacts that are introduced when converting data from one space to another (Klein et al., 2010a). However, surface-based labeling protocols avoid the use of cutting planes that arbitrarily cut through a volume to connect landmarks, and anecdotal evidence suggests that they require less time to learn and to apply consistently.

We created a surface-based cortical labeling protocol to set a new standard of labeling accuracy and consistency for use by the scientific community, as well as to create the largest and most complete set of labeled brains ever released to the public, called the “Mindboggle-101” dataset because of its concurrent development and use with the Mindboggle automated labeling and shape analysis software. In this article we introduce this dataset of manually edited brain image labels applied to the *T*1-weighted MR images of publicly available multi-modal data acquired from healthy individuals. We also introduce a benchmark for the evaluation of automated registration/segmentation/labeling methods by comparing the manual labels according to this “Desikan–Killiany–Tourville” (DKT) protocol with automatically generated labels. All data, software, and information related to this study will be available as a public resource on the http://mindboggle.info/data website under a Creative Commons license^3^.

## Materials and Methods

### Data

We selected 101 *T*1-weighted brain MR images that are: (1) publicly accessible with a non-restrictive license, (2) from healthy participants, (3) of high quality to ensure good surface reconstruction, and (4) part of a multi-modal acquisition (*T*2*-weighted, diffusion-weighted scans, etc.). Five subjects were scanned specifically for this dataset (MMRR-3T7T-2, Twins-2, and Afterthought-1). Scanner acquisition and demographic information are included as Supplementary Material and are also available on the http://mindboggle.info/data website. Table 1 lists the data sets that comprise the Mindboggle-101 data set. These include the 20 test–retest subjects from the “Open Access Series of Imaging Studies” data (Marcus et al., 2007), the 21 test–retest subjects from the “Multi-Modal Reproducibility Resource” (Landman et al., 2011), with two additional subjects run under the same protocol in 3T and 7T scanners, 20 subjects from the “Nathan Kline Institute Test–Retest” set, 22 subjects from the “Nathan Kline Institute/Rockland Sample”, the 12 “Human Language Network” subjects (Morgan et al., 2009), the Colin Holmes 27 template (Holmes et al., 1998), two identical twins (including author AK), and one brain imaging colleague.

**Table 1:** **Data sets comprising the Mindboggle-101 labeled data set**.

Name	Source	*N*	Age (mean, SD)	Gender		Hand	
				M	F	R	L
NKI-RS-22	“Nathan Kline Institute/Rockland sample”	22	20–40 (26.0, 5.2)	12	10	21	1
NKI-TRT-20	“Nathan Kline Institute/Test–retest”	20	19–60 (31.4, 11.1)	14	6	15*	3*
MMRR-21	“Multi-modal MRI reproducibility resource”	21	22–61 (31.8, 9.2)	11	10	18	1
MMRR-3T7T-2	2 3T/7T subjects acquired after the MMRR-21 subjects	2	22, 24	2	0	2	0
HLN-12	“Human language network” study subjects	12	23–39 (27.8, 4.6)	6	6	12	0
OASIS-TRT-20	“Open access series of imaging studies” test–retest (“reliability”) sample	20	19–34 (23.4, 3.9)	8	12	20	0
Colin27-1	Colin Holmes template	1	33	1	0	1	0
Twins-2	Two identical twins, incl. AK	2	41	2	0	2	0
Afterthought-1	Brain imager SG	1	36	1	0	1	0

*(2 ambidextrous)

NKI-RS-22 – http://fcon_1000.projects.nitrc.org/indi/pro/nki.html

NKI-TRT-24 – http://fcon_1000.projects.nitrc.org/indi/pro/eNKI_RS_TRT/FrontPage.html

MMRR-21 – http://www.nitrc.org/projects/multimodal

MMRR-3T7T-2 – http://www.nitrc.org/projects/multimodal

HLN-12 – https://masi.vuse.vanderbilt.edu/public/plos12.tar.bz2

OASIS-TRT-20 – http://www.oasis-brains.org/app/action/BundleAction/bundle/OAS1_RELIABILITY

Colin27-1 – http://www.bic.mni.mcgill.ca/ServicesAtlases/Colin27

We preprocessed and segmented *T*1-weighted MRI volumes and constructed cortical surfaces using FreeSurfer’s standard recon-all image processing pipeline^4^ (Dale et al., 1999; Fischl et al., 1999). Since it has been demonstrated recently that FreeSurfer results can vary depending on software version, operating system, and hardware (Gronenschild et al., 2012), every group of subjects was processed by FreeSurfer with the same computer setup. All images were run on Apple OSX 10.6 machines, except for two (Twins-2, run on Ubuntu 11.04), and all were run using FreeSurfer version 5.1.0, except for the OASIS-TRT-20, which were run using 5.0.0 (manual labeling was completed prior to the availability of v5.1.0). Following an initial pass, JT inspected segmentation and surface reconstructions for errors (manual edits to the gray–white tissue segmentation were required for a single subject: HLN-12-2). FreeSurfer then automatically labeled the cortical surface using its DK cortical parcellation atlas ([lh,rh].curvature.buckner40.filled.desikan_killiany.2007 06 20gcs for left and right hemispheres). Vertices along the cortical surface are assigned a given label based on local surface curvature and average convexity, prior label probabilities, and neighboring vertex labels (S’egonne et al., 2004; Desikan et al., 2006). The region definitions of the labeling protocol represented by the DK atlas are described in Desikan et al. (2006).

### Desikan–killiany–tourville labeling protocol

The goal of this work was to create a large dataset of consistently and accurately labeled cortices. To do so we adopted a modification of the DK protocol (Desikan et al., 2006). We modified the protocol for two reasons: (i) to make the region definitions as consistent and as unambiguous as possible, and (ii) to rely on region boundaries that are well suited to FreeSurfer’s classifier algorithm, such as sulcal fundi that are approximated by surface depth and curvature. This would make it easier for experienced raters to assess and edit automatically generated labels, and to minimize errors introduced by the automatic labeling algorithm. We also sought to retain major region divisions that are of interest to the neuroimaging community. In some cases, this necessitated the inclusion of anatomically variable sulci as boundary markers (such as subdivisions of the inferior frontal gyrus) or use of gyral crowns (such as the pericalarine cortex). Alternatively, common subdivisions of gyri that were not based on cortical surface curvature features (such as subdivisions of the cingulate gyrus and the middle frontal gyrus) were retained if the subdivision *was wholly within* the surface curvature features that defined the gyrus.

The DKT protocol has 31 cortical regions per hemisphere, one less than the DK protocol. We have also created a variant of the DKT protocol with 25 cortical regions per hemisphere to combine regions that are subdivisions of a larger gyral formation and whose divisions are not based on sulcal landmarks or are formed by sulci that are highly variable. The regions we combined include subdivisions of the cingulate gyrus, the middle frontal gyrus, and the inferior frontal gyrus. Since fewer regions means larger regions that lead to higher overlap measures when registering images to each other, note that comparisons should be made using the same labeling protocol. We refer to these two variants as the DKT31 and DKT25 cortical labeling protocols.

**Figure 1:**
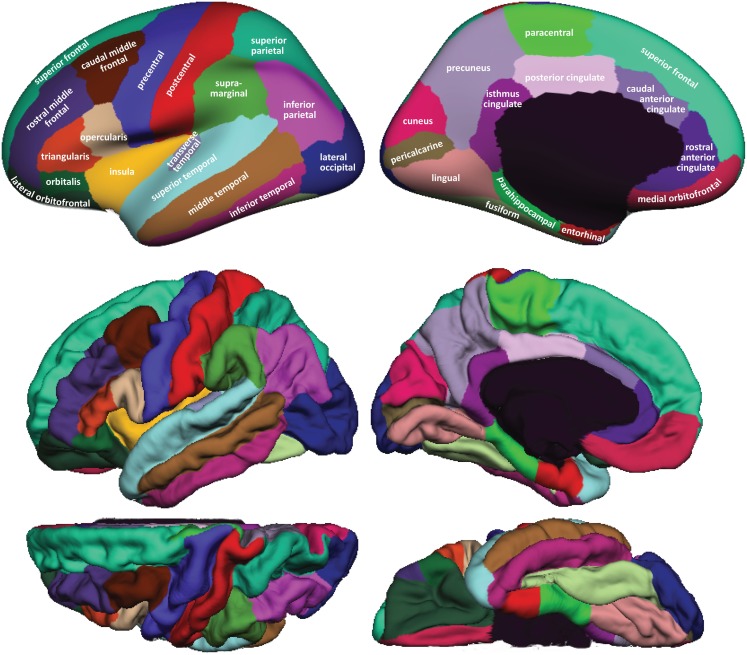
**Regions in the DKT cortical labeling protocol**. Cortical regions of interest included in the DKT protocol are displayed on the left hemisphere of the FreeSurfer “fsaverage” average brain template. *Top*: regions overlaid on lateral (left) and medial (right) views of the inflated cortical surface. The unlabeled area at the center of the medial view corresponds to non-cortical areas along the midline of the prosencephalon. *Bottom*: regions overlaid on lateral (upper left), medial (upper right), dorsal (lower left), and ventral (lower right) views of the pial surfaces. The surface was automatically labeled with the DKT40 classifier atlas then manually edited as needed. The “fsaverage” data are included in the FreeSurfer distribution in $FREESURFER_HOME/subjects/fsaverage and the DKT-labeled version is available at http://mindboggle.info/data.

**Figure 2:**
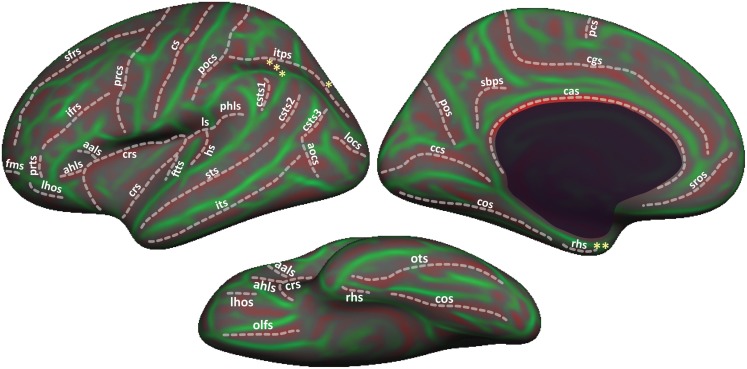
**Sulci in the DKT protocol**. Sulci that form the region boundaries are drawn and labeled on the inflated “fsaverage” left hemisphere lateral (top left), medial (top right), and ventral (bottom) cortical surface. A map of surface curvature is indicated by the red-green colormap. Convex curvature corresponding to gyral crowns are shown in green; concave curvature corresponding to sulcal fundi are shown in red. The masked area at the center of the medial view corresponds to non-cortical areas along the midline of the prosencephalon. “*”, “**”, and “***” indicate the approximate locations of the transverse occipital sulcus, the temporal incisure, and the primary intermediate sulcus, respectively. These landmarks are not clearly distinguishable on the “fsaverage” inflated surface.

Figure 1 shows cortical regions in the DKT labeling protocol. We retained the coloring scheme and naming conventions of Desikan et al. (2006) for ease of comparison. The Appendix contains detailed definitions of the regions but we summarize modifications to the original DK protocol in Table 2. Table 3 lists the names and abbreviations for the bounding sulci used by the DKT protocol; the locations of these sulci are demonstrated in Figure 2. Three regions were eliminated from the original DK protocol: the frontal and temporal poles and the banks of the superior temporal sulcus. The poles were eliminated because their boundaries were comprised primarily of segments that “jumped” across gyri rather than along sulci. By redistributing these regions to surrounding gyri we have increased the portion of region boundaries that along similar curvature values, that is, along sulci and gyri rather than across them, which improves automatic labeling and the reliability of manual edits. The banks of the superior temporal sulcus region was eliminated because its anterior and posterior definitions were unclear and it spanned a major sulcus.

**Table 2:** **Cortical regions in the DKT labeling protocol**.

**Temporal lobe *medial aspect***
Entorhinal cortex
Parahippocampal gyrus
Temporal pole* [removed]
Fusiform gyrus
**Temporal lobe (*lateral aspect*)**
Superior temporal gyrus
Middle temporal gyrus
Inferior temporal gyrus
Transverse temporal gyrus
Banks of superior temporal sulcus* [removed]
**Frontal lobe**
Superior frontal
Middle frontal gyrus*
Rostral
Caudal
Inferior frontal gyrus*
*Pars opercularis*
*Pars triangularis*
*Pars orbitalis*
Orbitofrontal gyrus
Lateral division
Medial division
Frontal pole* [removed]
Precentral gyrus
Paracentral lobule
**Parietal lobe**
Postcentral gyrus
Supramarginal gyrus
Superior parietal lobule
Inferior parietal lobule
Precuneus
**Occipital lobe**
Lingual gyrus
Pericalcarine cortex
Cuneus cortex
Lateral occipital cortex
Cingulate cortex*
Rostral anterior
Caudal anterior
Posterior
Isthmus

*An asterisk indicates a difference from the Desikan–Killiany protocol; see Appendix for more detailed descriptions*.

**Table 3:** **Sulci included in the DKT labeling protocol and their abbreviations**.

aals	Anterior ascending ramus of the lateral sulcus
ahls	Anterior horizontal ramus of the lateral sulcus
aocs	Anterior occipital sulcus
cas	Callosal sulcus
ccs	Calcarine sulcus
cgs	Cingulate sulcus
cos	Collateral sulcus
crs	Circular insular sulcus
cs	Central sulcus
csts1	Caudal superior temporal sulcus, first segment
csts2	Caudal superior temporal sulcus, second segment
csts3	Caudal superior temporal sulcus, third segment
fms	Frontomarginal sulcus
ftts	First transverse temporal sulcus
hs	Heschl’s sulcus
ifrs	Inferior frontal sulcus
ihs	Interhemispheric sulcus
itps	Intraparietal sulcus
its	Inferior temporal sulcus
lhos	Lateral H-shaped orbital sulcus
locs	Lateral occipital sulcus
ls	Lateral sulcus
mhos	Medial H-shaped orbital sulcus
olfs	Olfactory sulcus
ots	Occipitotemporal sulcus
pals	Posterior ascending ramus of the lateral sulcus
pcs	Paracentral sulcus
phls	Posterior horizontal ramus of the lateral sulcus
pis	Primary intermediate sulcus
pocs	Postcentral sulcus
pos	Parietooccipital sulcus
prcs	Precentral sulcus
prts	Pretriangular sulcus
rhs	Rhinal sulcus
sbps	Subparietal sulcus
sfrs	Superior frontal sulcus
sros	Superior rostral sulcus
sts	Superior temporal sulcus
ti	Temporal incisure
tocs	Transverse occipital sulcus

Additional, more minor, modifications took the form of establishing distinct sulcal boundaries when they approximated a boundary in the original protocol that was not clearly defined. For instance, the lateral boundary of the middle temporal gyrus anterior to the inferior frontal sulcus was defined explicitly as the lateral H-shaped orbital sulcus and the frontomarginal sulcus more anteriorly. Similarly, the boundary between the superior parietal and the lateral occipital regions was assigned to the medial segment of the transverse occipital sulcus. Other examples include establishing the rhinal sulcus and the temporal incisure as the lateral and anterior borders of the entorhinal cortex, and adding the first segment of the caudal superior temporal sulcus (Petrides, 2011) as part of the posterior border of the supramarginal gyrus. Several popular atlases informed these modifications, including Ono et al. (1990), Damasio (2005), Duvernoy (1999), and Mai et al. (2008). The recent sulcus and gyrus atlas from Petrides (2011) proved particularly useful because of its exhaustive catalog of small but common sulci.

### Label editing procedure

**Figure 3:**
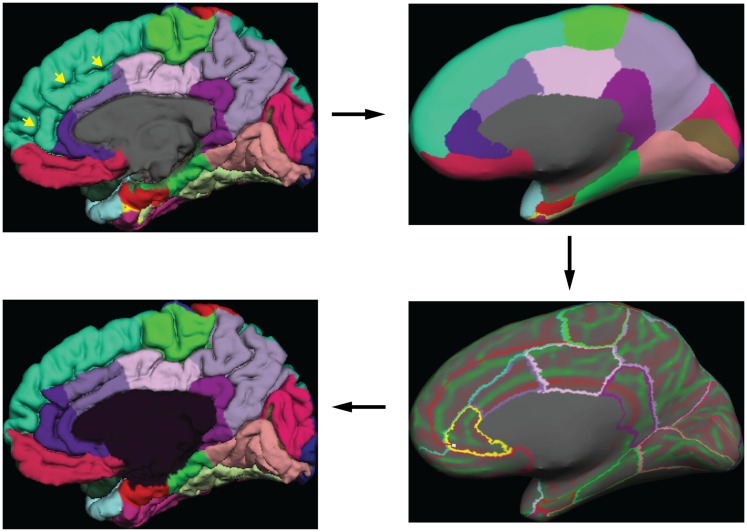
**Label editing example**. A typical manual edit is demonstrated. In the upper left, the pial surface of the right hemisphere is shown with labels generated from the DKT40 classifier atlas. Yellow arrowheads indicate a “double parallel” cingulate sulcus. The atlas failed to extend the rostral and caudal anterior cingulate regions dorso-rostrally to this sulcus, a common error when a parallel cingulate sulcus is present. To correct the error, the rater switches to the inflated surface view (upper right panel), and displays only the region outlines (lower right), which makes the cortical curvature map viewable. The rater then uses the curvature information to draw a line connecting vertices along the fundus of the parallel cingulate sulcus. Additional lines are drawn to subdivide the cingulate gyrus and the new regions are filled and labeled appropriately (bottom left panel). The yellow highlighted outline in the lower right panel indicates the last selected region (rostral anterior cingulate) and the light blue cursor mark within that region indicates the last selected surface vertex.

Greg Millington (GM) at Neuromorphometrics, Inc.^5^ edited the initial labels under the supervision of JT to ensure adherence to the DKT protocol. The editing procedure is outlined in Figure 3. GM relied on curvature maps overlaid on the native and inflated cortical (gray–white matter) surface and exterior cerebral (“pial”) surface to guide manual edits. JT inspected, and where necessary further edited, all manual edits. All manual edits were guided by the white matter, pial, and inflated surfaces, *and the T1-weighted volume*. While labeling was performed on the surface, we use topographical landmarks visible in the folded surface to infer label boundaries, so the volume remained the “ground truth” for evaluating anatomical decisions.

FreeSurfer’s DK classifier atlas assigned the initial labels for 54 of the brains in the Mindboggle-101 data set (OASIS-TRT-20, HLN-12, MMRR-21, and MMRR-3T7T-2). These were then manually edited by GM and JT to conform to the DKT protocol as described above. We selected the first 40 brains that we labeled (20 male, 20 female, 26 ± 7 years of age, from the MMRR-21, OASIS-TRT-20, and HLN-12 data) to train a new FreeSurfer cortical parcellation atlas representing the DKT protocol (see http://surfer.nmr.mgh.harvard.edu/fswiki/FsTutorial/GcaFormat; S’egonne et al., 2004; Desikan et al., 2006 for details regarding the algorithm that generates the atlas and how it is implemented). The resulting “DKT40 classifier atlas” then automatically generated the initial set of cortical labels for the remaining 47 brains in the data set (see http://surfer.nmr.mgh.harvard.edu/fswiki/mris_ca_label). To our knowledge, the DKT40 atlas was generated in the same manner as the DK atlas except for differences in the labeling protocol and training set.

### Comparison of manually edited and automated labels

To set a benchmark for the evaluation of future automated registration, segmentation, and labeling methods, we computed the volume overlap between each manually labeled region in each of 42 subjects (NKI-RS-22 and NKI-TRT-20) and the corresponding automatically labeled region (in the same subject) generated by two automated labeling methods. The overlap measure was the Dice coefficient (equal to the intersection of the two regions divided by their average volume) and was computed after propagating the surface labels through the subject’s gray matter mask (using the command mri_aparc2aseg). For the first automated labeling method, we used FreeSurfer’s automated parcellation software once with the DK classifier atlas and separately with our DKT40 classifier atlas. The second method was a multi-atlas approach that registered multiple atlases to each subject. First we constructed two average FreeSurfer templates, one for the NKI-RS-22 group and the other for the NKI-TRT-20 group. We then used FreeSurfer’s surface-based registration algorithm to register all of the manually labeled NKI-RS-22 surfaces to each of the NKI-TRT-20 surfaces via the NKI-TRT-20 template, and likewise registered all of the manually labeled NKI-TRT-20 surfaces to each of the NKI-RS-22 surfaces via the NKI-RS-22 template. For each surface vertex in each subject, we then assigned a single label from the multiple registered labels by majority-vote rule, resulting in a set of maximum probability or majority-vote labels for each subject. The Python software for performing the multi-atlas labeling is available on the website: http://www.mindboggle.info/papers.

## Results

**Figure 4:**
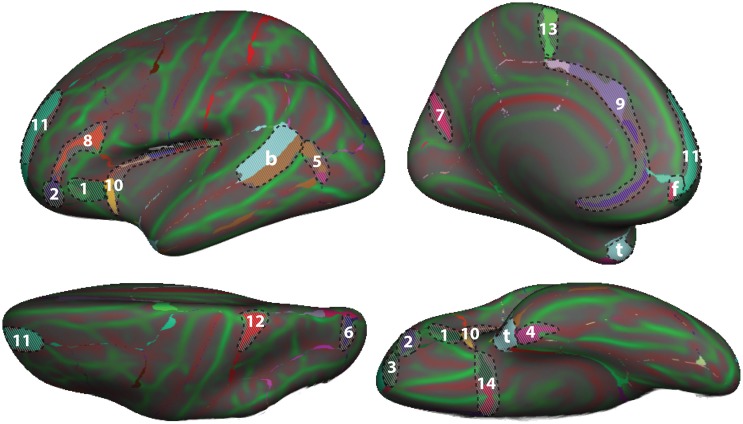
**Comparison of DK and DKT40 classifier atlases**. A comparison of the automatic labeling of the FreeSurfer “fsaverage” cortical surface by the DK and DKT40 atlases. Lateral (*upper left*), medial (*upper right*), ventral (*lower right*), and dorsal (*lower left*) views of the left hemisphere surface are shown. Regions in color overlaid atop the red-green surface (as in Figure 2) indicate areas that were labeled differently by the classifiers; where there are mismatches, the DKT40 labels are shown (with the same colors as in Figure 1). Areas denoted by letters mark the approximate location of regions in the DK protocol that were removed in the DKT protocol, including the banks of the superior temporal sulcus (b), frontal pole (f), and temporal pole (t). Additional, relatively large mismatched areas are denoted by numbers. Sources of mismatch between the protocols include: i, differences in region boundaries, particularly for the medial (1) and anterior (2) borders of *pars orbitalis*, the anterior border of the lateral orbitofrontal region (3), the lateral border of entorhinal cortex (4), and the anterior boundary of lateral orbital gyrus (5), and the posterior boundary of the superior parietal region (6); ii, variability of the bounding landmarks, particularly for the fundus of the parietooccipital sulcus (7) and the inferior frontal sulcus (8); iii, variation in the interpretation of landmarks, particularly for the cingulate sulcus (9), dorso-rostral portion of the circular sulcus (10), the rostral portion of superior frontal sulcus (11), the dorsal portion of the postcentral sulcus (12), the paracentral sulcus (13), and the posterior boundary of the medial and lateral orbitofrontal regions (14), and iv, variation in the training data set that was used to construct the classifier. The medial surface view was rotated from the parasagittal plane to expose the temporal pole.

To demonstrate differences between the DK and DKT40 classifier atlases, we used both to label the Freesurfer “fsaverage” cortical surface template. Figure 4 shows mismatches between the automatically generated labels for the left hemisphere surface. In addition to differences associated with the removal of regions from the DK protocol (areas denoted by letters in Figure 4), several other areas of mismatch are notable (areas denoted by numbers). Mismatches in these areas are due to a number of sources, including changes in the boundary definitions of regions common to both protocols, high variability of some common region boundary landmarks, and variation in the interpretation of bounding landmarks. While there may be a primary cause of differences between the atlases, the mismatched areas shown in Figure 4 may be due to any combination of these factors. An additional source of variability contributing to the mismatch areas is the reliance on different training datasets for the construction of the two atlases. While there are several areas of mismatch, including large portions of some regions, the overall overlap of labels generated by the two classifier atlases was high: overall Dice overlap was 89% in the left hemisphere and 90% in the right hemisphere.

Table 4 contains Dice overlap measures computed for each manually and automatically labeled cortical region, averaged across all of the 42 Nathan Kline subjects (NKI-RS-22 and NKI-TRT-20). The overlaps are higher than those computed in a prior study (Klein et al., 2010b, Table 3 in Supplementary Material) which performed a single-atlas version of the multi-atlas labeling, confirming that it is better to use multiple atlases. Only the DKT and multi-atlas overlap values were generated using the same atlas brains following the DKT labeling protocol, so these values may be directly compared with one another (and not with the DK values). According to a Wilcoxon signed-rank test, the DKT mean overlap values are significantly greater than the multi-atlas mean overlap values (*p* < 10^−10^).

**Table 4:** **Overlap results of manually and automatically labeled cortical regions**.

	DK atlas	DKT atlas	Multi-atlas
**TEMPORAL LOBE**			
L entorhinal	81.85 (10.18)	86.24 (9.94)	85.38 (6.51)
R entorhinal	77.15 (13.68)	81.98 (14.58)	81.16 (10.71)
L parahippocampal	93.76 (4.98)	95.71 (5.23)	91.21 (3.90)
R parahippocampal	93.05 (6.38)	95.28 (6.88)	91.64 (4.91)
L fusiform	83.55 (5.41)	89.46 (4.64)	87.89 (3.48)
R fusiform	83.76 (5.71)	88.85 (5.41)	87.51 (5.60)
L superior temporal	82.35 (3.85)	95.00 (2.55)	94.34 (2.47)
R superior temporal	81.53 (3.63)	93.71 (3.03)	93.38 (2.78)
L middle temporal	83.55 (4.33)	91.43 (3.75)	89.75 (3.43)
R middle temporal	86.72 (4.64)	92.40 (4.67)	91.17 (3.82)
L inferior temporal	81.11 (7.47)	89.17 (5.23)	87.05 (4.06)
R inferior temporal	85.95 (6.02)	90.09 (4.86)	88.37 (4.42)
L transverse temporal	94.48 (5.17)	95.63 (5.76)	92.50 (4.58)
R transverse temporal	90.22 (8.85)	91.05 (9.44)	87.11 (7.63)
**FRONTAL LOBE**			
L superior frontal	83.08 (4.92)	90.23 (4.64)	89.82 (3.60)
R superior frontal	83.12 (4.58)	89.81 (6.10)	89.49 (4.31)
L rostral middle frontal	80.66 (4.96)	88.76 (5.77)	88.48 (4.19)
R rostral middle frontal	81.26 (5.45)	86.21 (7.56)	87.64 (3.25)
L caudal middle frontal	92.86 (5.30)	95.11 (5.53)	89.51 (6.17)
R caudal middle frontal	92.67 (4.92)	95.21 (5.41)	89.50 (6.25)
L pars opercularis	84.72 (7.18)	87.17 (7.96)	85.00 (9.08)
R pars opercularis	81.06 (9.69)	82.80 (10.02)	83.96 (9.38)
L pars triangularis	77.13 (7.55)	84.81 (9.50)	84.10 (9.26)
R pars triangularis	75.33 (10.06)	76.57 (12.85)	76.72 (11.20)
L pars orbitalis	51.37 (13.16)	82.79 (15.48)	81.26 (15.00)
R pars orbitalis	54.68 (13.56)	74.50 (13.32)	70.25 (11.17)
L lateral orbitofrontal	82.65 (3.38)	93.12 (2.84)	93.16 (2.28)
R lateral orbitofrontal	83.63 (2.87)	94.23 (2.85)	93.53 (2.33)
L medial orbitofrontal	80.75 (7.02)	91.37 (5.93)	90.09 (4.60)
R medial orbitofrontal	76.40 (8.01)	90.78 (5.35)	89.31 (3.17)
L precentral	94.62 (2.93)	96.22 (3.06)	92.99 (4.42)
R precentral	93.40 (3.60)	96.52 (3.80)	93.63 (3.91)
L paracentral	86.92 (5.43)	92.67 (6.21)	89.24 (7.06)
R paracentral	90.10 (5.14)	92.19 (5.62)	89.67 (5.56)
**PARIETAL LOBE**			
L postcentral	89.63 (4.26)	93.52 (4.25)	89.73 (5.99)
R postcentral	91.53 (2.80)	96.07 (3.13)	93.04 (2.85)
L supramarginal	88.38 (5.03)	89.84 (5.63)	87.39 (4.69)
R supramarginal	87.99 (4.02)	89.59 (4.04)	87.08 (5.04)
L superior parietal	85.29 (3.58)	92.64 (3.29)	87.94 (4.68)
R superior parietal	85.06 (4.60)	91.31 (4.88)	89.03 (4.67)
L inferior parietal	87.81 (5.51)	89.93 (5.91)	87.41 (5.70)
R inferior parietal	88.21 (4.60)	89.83 (4.93)	88.26 (4.03)
L precuneus	93.05 (2.31)	96.26 (2.79)	93.04 (3.02)
R precuneus	91.38 (2.88)	95.00 (3.33)	92.19 (2.61)
**OCCIPITAL LOBE**			
L lingual	96.93 (2.42)	97.71 (2.46)	95.12 (2.94)
R lingual	97.01 (3.89)	97.91 (4.04)	95.15 (3.66)
L pericalcarine	95.78 (5.11)	97.40 (5.20)	92.75 (4.80)
R pericalcarine	95.44 (4.83)	96.99 (4.88)	91.75 (4.23)
L cuneus	80.33 (5.19)	93.64 (5.58)	89.63 (5.41)
R cuneus	80.35 (7.59)	90.06 (7.38)	85.24 (7.32)
L lateral occipital	87.79 (4.07)	90.40 (4.17)	88.18 (3.35)
R lateral occipital	85.94 (5.72)	88.05 (5.97)	87.27 (5.13)
**CINGULATE CORTEX**			
L rostral ant. cingulate	82.34 (8.42)	88.23 (10.15)	85.74 (7.92)
R rostral ant. cingulate	81.12 (13.42)	86.78 (13.40)	82.95 (11.53)
L caudal ant. cingulate	72.96 (16.62)	87.80 (7.40)	68.13 (7.06)
R caudal ant. cingulate	83.52 (15.49)	85.69 (14.86)	63.50 (9.82)
L post. cingulate	91.69 (6.25)	95.57 (5.92)	93.25 (4.55)
R post. cingulate	92.19 (5.91)	95.87 (5.02)	93.74 (4.53)
L isthmus cingulate	93.21 (3.84)	94.93 (4.00)	93.56 (3.80)
R isthmus cingulate	90.82 (15.00)	92.68 (15.24)	90.11 (14.93)
L insula	88.82 (1.50)	98.51 (1.29)	97.72 (0.93)
R insula	89.57 (2.68)	98.77 (1.29)	97.77 (0.87)

*All numbers are Dice overlap measures between manual and automated labels, averaged (with SD) across 42 (NKI-RS-22 and NKI-TRT-20) subjects. Automated labels were generated using FreeSurfer’s DK classifier atlas, our DKT40 classifier atlas, and by multi-atlas registration. In no case was the atlas used to label a participant’s brain image constructed using that participant’s brain image. In multi-atlas registration, each of the labeled brains used to construct the DKT40 classifier atlas was registered to a subject via an average surface template, resulting in multiple labels for each vertex of the subject’s surface, and a single label was chosen by majority-vote rule. Overlap was computed after propagating the surface labels through the subject’s gray matter mask. Since the DK atlas column uses an atlas constructed from non-Mindboggle-101 image data and uses a different labeling protocol than the other two columns, the overlap measures are not intended to be compared with the other columns but may instead be considered benchmark overlap measures when using the standard FreeSurfer atlas. Likewise, the DKT40 atlas and multi-atlas columns may be used as benchmark overlap measures when using the DKT40 atlas or multiple Mindboggle-101 atlases. According to a Wilcoxon signed-rank test, the DKT40 atlas values are significantly greater than the multi-atlas values (*p* < 10^−10^)*.

The Dice values are in general very high for the DKT auto/manual comparison (mean: 91 ± 6, range: 74–99). The Dice values are lower for regions that rely on anatomically variable sulci and when the region is bounded by discontinuous surface features. The *pars orbitalis* and *pars triangularis*, which had the lowest Dice coefficients, are affected by both factors. These relatively small regions are divided by the anterior horizontal ramus of the lateral sulcus. The length and location of this sulcus varies greatly with respect to nearby landmarks. Their anterior border is formed, in part, by another small, variable sulcus, the pretriangular sulcus. However, this sulcus rarely forms the entire anterior border of either region. Rather, the division between these regions and the more anterior middle frontal gyrus typically requires “jumps” across gyri. This makes reliable labeling of this region difficult for both an automatic algorithm and an experienced rater. A counter example is the insula (Dice > 98%) which is surrounded by a consistent, easily identified sulcus. Overlap measures are also biased in favor of larger regions.

## Discussion

In this article, we introduced the largest and most complete set of free, publicly available, manually labeled human brain images – 101 human cortices labeled according to a new surface-based cortical labeling protocol. These data are available^6^ under a Creative Commons (attribution-non-commercial-sharealike 3.0) license (see text footnote 3). We compared the manual labels with labels generated by automated labeling methods to set benchmarks for the evaluation of automated registration/labeling methods.

Any automated labeling method could be used to initialize the labels for further editing by a human. We chose FreeSurfer for this study because it performs registration-based labeling well (Klein et al., 2010b), its classifier uses similar geometric properties such as local curvature that our DKT protocol follows, and because it offers a good interface for editing labels. And while the automated labeling algorithm turns the problem into one that is machine-assisted or semi-automatic, we use our manual labeling in turn to improve the automated labeling, in this study by creating a new classifier atlas. Our next step is to modify the current protocol to further improve the reliability and accuracy of both automatic and manual labeling. For instance, highly variable boundaries may be replaced or eliminated, resulting in the aggregation of existing labels (as in the DKT25 vs. DKT31 protocol). Alternatively, the experience of reviewing the cortical topography of such a large number of brains in a relatively short period of time has made apparent the existence of additional robust cortical features. For instance, the “temporo-limbic gyral passage” (Petrides, 2011) is commonly observed in the basal temporal area. Adding this relatively small region to the protocol will make labeling this area of the brain more straightforward. We have also begun to use automatically extracted cortical features to refine these manual labels so that they follow stringent guidelines for curvature and depth, a difficult task for a human rater. Even without these aids, we were able to reduce the time required to label cortical regions to under 2 hours/brain of an experienced human rater’s time. Thus we are now able to label 10 or more brains in the time that one could be labeled fully manually, and with a similar level of accuracy.

Our original purpose for the Mindboggle-101 dataset was to create a publicly accessible online morphometry database to study anatomical variation, and for widespread use in training and testing automated algorithms. Other future goals include labeling more brain images from different demographic and clinical populations, creating more optimal average templates (Avants et al., 2010) and probabilistic atlases based on these data, and incorporating what we learn from future labeling efforts into future versions of the labeling protocol (updates will be posted on http://mindboggle.info/data).

## Author Contributions

Arno Klein directed the labeling as part of the Mindboggle project, wrote and applied software for multi-atlas labeling, surface template construction, and evaluation of labels based on volume overlap measures, and wrote the manuscript. Jason Tourville created the cortical labeling protocol, supervised the manual labeling, performed the final labeling edits and approvals, constructed a new FreeSurfer atlas with some of the labeled data, and contributed to the writing of the manuscript.

## Conflict of Interest Statement

The authors declare that the research was conducted in the absence of any commercial or financial relationships that could be construed as a potential conflict of interest.

## Supplementary Material

The Supplementary Material for this article can be found online at http://www.frontiersin.org/Brain_Imaging_Methods/10.3389/fnins.2012.00171/abstract

## Footnotes

^1^http://www.loni.ucla.edu/Protocols/

^2^http://www.braincolor.org

^3^http://creativecommons.org/licenses/by-nc-sa/3.0/deed.en_US

^4^http://surfer.nmr.mgh.harvard.edu/

^5^http://www.neuromorphometrics.com

^6^http://mindboggle.info/data/

## Appendix

### Cortical region definitions

This labeling protocol is an adaptation of the DK cortical region definitions (Desikan et al., 2006). Abbreviations below are for *sulci* (see Table 3) and anatomical terms of location:

A: anterior; P: posterior; V: ventral; D: dorsal; M: medial; L: lateral.

### Temporal lobe, *medial aspect*

#### Entorhinal cortex

A: temporal incisure (rostral limit of *cos*); P: posterior limit of the amygdala; D: medio-dorsal margin of the temporal lobe anteriorly, amygdala posteriorly; M: *rhs* (*cos*), or the *cos* if the *rhs* is not present.

#### Parahippocampal gyrus

A: posterior limit of the amygdala; P: posterior limit of the hippocampus; D: hippocampus; M: *cos*.

#### Temporal pole [removed]

The area included in the DK temporal pole has been redistributed to the superior, middle, and inferior temporal gyrus regions.

#### Fusiform gyrus

A: anterior limit of *ots* (anterior limit of *cos*); P: first transverse sulcus posterior to the temporo-occipital notch (rostral limit of the superior parietal gyrus); M: *cos*; L: *ots*.

### Temporal lobe, *lateral aspect*

#### Superior temporal gyrus

A: anterior limit of the *sts* or a projection from the *sts* to the anterior limit of the temporal lobe; P: junction of *phls* (or its posterior projection) and caudal *sts* (*csts1*, *2*, or *3*); M: The superior temporal gyrus includes the entire ventral bank of the *ls* with the exception of the transverse temporal gyrus, and therefore has a varying medial boundary. Listed from anterior to posterior they include, anterior to the temporo-frontal junction, the medial margin of the temporal lobe or the entorhinal cortex (posterior to the temporal incisures); *crcs* anterior to *ftts*; the dorsolateral margin of the temporal lobe between anterior limits of *ftts* and *hs*; *hs* anterior to the junction of *hs* and *crcs*; *lh* anterior to *phls*; *phls*; L: *sts*.

#### Middle temporal gyrus

A: anterior limit of *sts*, P: *aocs*, M: *sts* anteriorly, posteriorly formed by *csts3*; L: its.

#### Inferior temporal gyrus

A: anterior limit of *ifs*; P: *aocs*; M: *ots*; L: *ifs*.

#### Transverse temporal gyrus

A: anterior limit of *ftts*; P: posterior limit of *hs*; M: *ftts*; L: *hs*; between the anterior limits of *ftts* and *hs*, the lateral boundary is formed by the dorsolateral margin of the temporal lobe.

#### Banks of superior temporal sulcus [removed]

The area in the banks of the superior temporal sulcus in the DK protocol has been redistributed to the superior and middle temporal gyri.

### Frontal lobe

#### Superior frontal gyrus

A: *fms*; P: *prcs* (lateral surface); *pcs* (medial surface); M: *cgs*, *sros* anterior to anterior limit of *cgs*; L: *sfs*.

#### Middle frontal gyrus

A: anterior limit of *sfs*; P: *prcs*; M: *sfs*; L: *ifs*; anterior to *ifs*, the ventro-lateral boundary is formed by *fms* and *lhos*.

The DK protocol divides the middle frontal gyrus into *rostral* and *caudal* subdivisions. The posterior limit of the *ifs* represents the approximate bounding landmark between the two regions. These regions remain in the DKT protocol; the division between the two was modified only if necessitated by changes in bounding sulci.

#### Inferior frontal gyrus, *pars opercularis*

A: *aals*; P: *prcs*; M: *ifs*; L: *crcs*.

#### Inferior frontal gyrus, *pars triangularis*

A: *prts*; P: *aals*; M: *ifs*; L: *ahls*; if *ahls* does not extend anteriorly to *prts*, an anterior projection from *ahls* to *prts* completes the lateral boundary.

#### Inferior frontal gyrus, *pars orbitalis*

A: *prts* – if *prts* does not extend ventrally to the *lhos*, a ventral projection from *prts* to *lhos* completes the anterior boundary; P: posterior limit of orbitofrontal cortex; M: *ahls* – if *ahls* does not extend anteriorly to *prts*, an anterior projection from *ahls* to *prts* completes the lateral boundary; L: *lhos*.

#### Orbitofrontal, lateral division

A: *fms*; P: posterior limit of orbitofrontal cortex; M: *ofs*; L: *lhos*.

#### Orbitofrontal, medial division

A: *fms*; P: posterior limit of orbitofrontal cortex; M: *sros*; if *sros* merges with *cgs*, the medial/dorsal boundary is formed by *cgs*; L: *ofs*.

#### Frontal pole [removed]

The area included in the DK frontal pole region has been redistributed to the superior frontal and orbitofrontal gyri.

#### Precentral gyrus

A: *prcs*; P: *cs*; M: dorsomedial hemispheric margin; L: *crcs*.

#### Paracentral lobule

A: *pcs*; P: marginal ramus of *cgs*; V: *cgs*; D: dorsomedial hemispheric margin.

### Parietal lobe

#### Postcentral gyrus

A: *cs*; P: *pocs*; M: dorsomedial hemispheric margin; L: *crcs* – if the lateral limit of *pocs* extends anterior to *crcs*, the posterior portion of the lateral/ventral boundary is formed by the lateral sulcus.

#### Supramarginal gyrus

A: *pocs*; P: the supramarginal gyrus is formed by sulci demarcating the cortical convolution surrounding the *pals* – posteriorly, this is typically formed by *pis* medially, and *csts1* laterally; M: *itps*; L: *ls* anterior to *phls*, *phls* posteriorly.

#### Superior parietal

A: *pocs*; P: tranverse sulcus lying immediately posterior to the *pos* – this is described as the *tocs*, medial segment, by Petrides (2011); M: dorsomedial hemispheric margin; L: *itps*.

#### Inferior parietal

A: *csts1*; P: junction of *locs* and *itps*; M: *itps*; L: *locs* anteriorly, *tocs*, lateral segment, posteriorly.

#### Precuneus

A: marginal ramus of *cgs* dorsally, *sbps* ventrally; P: *pos*; V: *ccs*; D: dorsomedial hemispheric margin.

### Occipital lobe

#### Lingual gyrus

A: posterior limit of the hippocampus; P: posterior limit of *ccs*; M: *ccs* anteriorly, medial limit of the ventral bank of *ccs* posterior to the junction of *pos* and *ccs*; L: *cos*.

#### Pericalcarine cortex

A: junction of *ccs* and *pos*; P: posterior limit of *ccs*; D: dorsomedial margin of *ccs*; V: ventromedial margin of *ccs*.

#### Cuneus cortex

A: *pos*; P: posterior limit of *ccs*; V: dorsomedial margin of *ccs*; D: dorsomedial hemispheric margin.

#### Lateral occipital cortex

A: temporo-occipital notch laterally, *aocs* more medially, *tocs*, medial segment, medial to *itps*; P: posterior limit of the occipital lobe; M: *locs* anteriorly; *tocs*, lateral segment, posteriorly; L: *ots* anteriorly; *cos* posterior to the transverse sulcus that marks the posterior limit of the fusiform gyrus; posterior to the posterior limit of *cos*, the lateral occipital cortex includes occipital cortex in its entirety.

#### Cingulate cortex

A/D: *cgs*; in the event of a “double parallel cingulate,” (e.g., Ono et al., 1990), the A/D boundary of the cingulate is formed by the more anterior-dorsal branch of the *cgs*; P: *sbps*; V: The cingulate gyrus is formed by the cortical convolution that lies dorsal to the corpus callosum, extending antero-ventrally around the *cc* genu and postero-ventrally around the *cc* splenium. Dorsal to the corpus callosum, the ventral boundary is formed by the *cas*. In the subgenual area, it is formed by the *cgs* and in the subsplenial area by the *ccs*. The DK atlas subdivides the cingulate gyrus into the subdivisions listed below from anterior to posterior locations with their bounding landmarks. These regions were left in the current protocol. Divisions between these regions were modified only if necessitated by changes in bounding sulci.

#### Rostral anterior

A: *cgs*; P: *cc* genu.

#### Caudal anterior

A: *cc* genu; P: mammillary bodies.

#### Posterior

A: mammillary bodies; P: junction of the *sbps* and *cgs* (approximately).

#### Isthmus

A: junction of the *sbps* and *cgs* (approximately); P: *sbps*.
